# Rapamycin up-regulates triglycerides in hepatocytes by down-regulating Prox1

**DOI:** 10.1186/s12944-016-0211-x

**Published:** 2016-02-27

**Authors:** Sora Kwon, Ji-Sook Jeon, Su Bin Kim, Young-Kwon Hong, Curie Ahn, Jung-Suk Sung, Inho Choi

**Affiliations:** Department of Pharmaceutical Engineering, Hoseo University, Asan, 336-795 Republic of Korea; Department of Surgery, Norris Comprehensive Cancer Center, Keck School of Medicine, University of Southern California, Los Angeles, CA USA; Transplantation Research Institute, Seoul National University, Seoul, Republic of Korea; Department of Life Science, Dongguk University, Goyang, 410-820 Republic of Korea

**Keywords:** Prospero-related homeobox 1, Triglycerides, Hepatocytes, Rapamycin

## Abstract

**Background:**

Although the prolonged use of rapamycin may cause unwanted side effects such as hyperlipidemia, the underlying mechanism remains unknown. Prox1 is a transcription factor responsible for the development of several tissues including lymphatics and liver. There is growing evidences that Prox1 participates in metabolism in addition to embryogenesis. However, whether Prox1 is directly related to lipid metabolism is currently unknown.

**Methods:**

HepG2 human hepatoma cells were treated with rapamycin and total lipids were analyzed by thin layer chromatography. The effect of rapamycin on the expression of Prox1 was determined by western blotting. To investigate the role of Prox1 in triglycerides regulation, siRNA and overexpression system were employed. Rapamycin was injected into mice for 2 weeks and total lipids and proteins in liver were measured by thin layer chromatography and western blot analysis, respectively.

**Results:**

Rapamycin up-regulated the amount of triglyceride and down-regulated the expression of Prox1 in HepG2 cells by reducing protein half-life but did not affect its transcript. The loss-of-function of Prox1 was coincident with the increase of triglycerides in HepG2 cells treated with rapamycin. The up-regulation of triglycerides by rapamycin in HepG2 cells reverted to normal levels by the compensation of Prox1 using the overexpression system. Rapamycin also down-regulated Prox1 expression but increased triglycerides in mouse liver.

**Conclusion:**

This study suggests that rapamycin can increase the amount of triglycerides by down-regulating Prox1 expression in hepatocytes, which means that the mammalian target of rapamycin (mTOR) signaling is important for the regulation of triglycerides by maintaining Prox1 expression.

## Background

Prospero-related homeobox 1 (Prox1) is a transcription factor responsible for the development of several tissues, such as lymphatics and liver [1–3]. *Prox1* haploinsufficient mice show lymphatic vascular defects leading to adult-onset obesity through the enhancement of adipogenesis and increased fat storage in lymphatic-rich regions. *Prox1* knock-out mouse embryos lack lymphatic systems and perish at day 14.5 of embryogenesis [4]. Prox1 is also known to regulate the activity of a specific subset of nuclear receptors including hepatocyte nuclear factor 4a (HNF4a, NR2A1) and liver receptor homolog-1 (LRH-1, NR5A2), which suggests that Prox1 may play a key role in the regulation of metabolism in the liver [5–7]. However, whether Prox1 directly participates in the regulation of lipid metabolism is currently unknown.

Rapamycin, also known as sirolimus, achieves its unique effects by binding to the mammalian target of rapamycin (mTOR), also known as FKBP12 rapamycin associated protein (FRAP) or rapamycin and FKBP12 target (RAFT). mTOR is a kind of serine-threonine kinase that belongs to the phosphatidylinositol (PI) kinase-related protein kinase family [8, 9]. It regulates cell growth and proliferation through translational control of several proteins such as cyclin dependent kinase inhibitor p27^kip1^, retinoblastoma protein, cyclin D1, c-myc and STAT 3 [10]. mTOR can be activated by several stimuli such as growth factors and nutrients through receptor tyrosine kinase (RTK), phosphatidyl inositol 3 kinase (PI3K), and Akt/PKB signaling cascade [11]. mTOR elicits its effect by binding to the cytosolic immunophilin FKBP12 (FK506 binding protein, 12kd) [12]. Due to its immunosuppressive properties, rapamycin has been used extensively in transplantation to prevent organ rejection [8, 13, 14]. Recently, clinical application of rapamycin has expanded to cancer therapy [15] as well as preventing occlusion of coronary arteries after stent placement [16]. In spite of rapamycin’s broad clinical application, it has been reported that prolonged use of rapamycin is associated with serious adverse effects, including hyperlipidemia [17–19]. Actually, rapamycin-associated dyslipidemia has been reported in 45 % of liver transplant patients [20] and in about 40 % of renal transplant patients [21]. These results suggest that, physiologically, mTOR signaling may play a significant role in lipid homeostasis.

In this study, we found that rapamycin increased the amount of triglycerides and down-regulated Prox1 expression in hepatocytes, which means that mTOR signaling is important for maintaining triglycerides as well as Prox1 expression. To the best of our knowledge, this study is the first report showing the down-regulation of Prox1 by the inhibition of mTOR signal and suggests that triglycerides are up-regulated by rapamycin through the down-regulation of Prox1.

## Results

### Rapamycin increases triglycerides in HepG2 cells

**Fig. 1:**
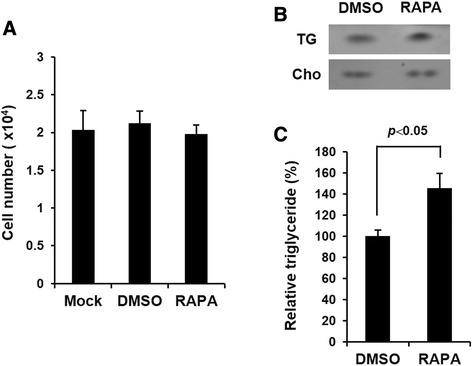
Rapamycin increases the amount of triglycerides in HepG2 cells but does not affect cellular proliferation. **a** HepG2 cells were cultured in low-serum media (1 % FBS) with or without rapamycin (RAPA, 10 nM). After 48 h, cell proliferation was measured by WST-1 assay. Mock: untreated control group. **b** HepG2 cells treated with or without rapamycin were harvest after 48 h and intracellular triglycerides was measured by thin layer chromatography (TLC). TG: triglycerides, Cho: cholesterol. **c** Relative triglycerides were analyzed using the Image J program (*p* < 0.05)

To investigate the physiological effect of rapamycin on hepatocytes, we first looked at the proliferation of HepG2 cells treated with rapamycin (RAPA). Compared with cells not treated with any drug (Mock) or treated with vehicle (DMSO) alone, rapamycin did not affect the proliferation of HepG2 cells (Fig. 1a). We then measured the amount of triglycerides in HepG2 cells by thin layer chromatography (TLC) and found that rapamycin increased the amount of triglycerides in HepG2 cells (Fig. 1b). In comparison, the quantity of intracellular cholesterol was constant across samples, but the relative triglycerides increased significantly in quantity in rapamycin-treated cells (Fig. 1c). These results indicate that rapamycin augments triglycerides in HepG2 cells although it does not affect liver cell proliferation.

### Rapamycin down-regulates Prox1 in HepG2 cells by decreasing protein stability

**Fig. 2:**
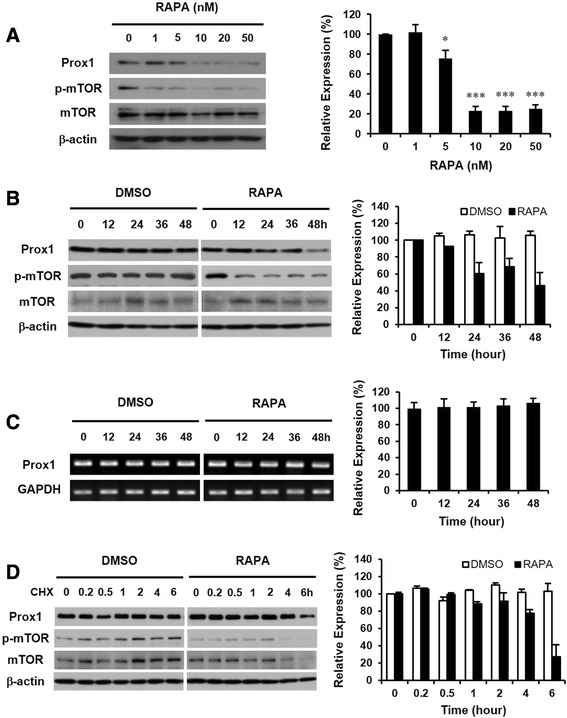
Rapamycin affects Prox1 protein but not its transcript. **a** HepG2 cells were treated with various concentrations of rapamycin from 1 to 50 nM. After 48 h, cells were harvested and analyzed by western blot. Phosphorylated mTOR (p-mTOR) was detected to confirm the inhibitory effect of rapamycin. mTOR and β-actin were measured as quantitative controls. (*: *p* < 0.05, ***: *p* < 0.001). **b** The expression of Prox1 in HepG2 cells was down-regulated by rapamycin after 24 h and decreased at 48 h. **c** Total RNAs were prepared from HepG2 cells incubated with or without rapamycin and analyzed by semi-quantitative RT-PCR (right panel) and real-time quantitative RT-PCR (left panel). **d** HepG2 cells were treated with or without rapamycin for 12 h and exposed to additional incubation with cycloheximide. The expression of Prox1 was maintained at 6 h after cycloheximide treatment in DMSO-treated HepG2 cells, but it decreased after 4 h in rapamycin-treated cells

Next we focused on the expression of Prox1 in HepG2 cells treated with rapamycin. First, we treated HepG2 cells with various concentrations of rapamycin from 1 to 50 nM. After 48 h, we analyzed Prox1 and found that its expression was decreased by rapamycin (Fig. 2a). The phosphorylated form of mTOR was detected to verify the inhibitory effect of rapamycin. Then we investigated the change in Prox1 expression induced by rapamycin over time. Although expression of *Prox1* gene in HepG2 cells was not affected by the vehicle (DMSO), it was down-regulated by rapamycin after 24 h and decreased at 48 h (Fig. 2b). We then investigated whether the down-regulation of Prox1 expression by rapamycin is occurred by affecting transcript or protein. We first checked *Prox1* mRNA in HepG2 cells and found that rapamycin does not affect the expression of *Prox1* mRNA (Fig. 2c). Then we employed cycloheximide to examine the half-life of the protein. Prox1 protein was expressed well at 6 h after cycloheximide treatment in DMSO-treated HepG2 cells, but it was decreased after 4 h in rapamycin-treated cells (Fig. 2d). These data demonstrate that rapamycin down-regulates Prox1 expression and affects protein stability but not mRNA expression.

### Prox1 is a critical factor for the regulation of triglycerides in HepG2 cells

**Fig. 3:**
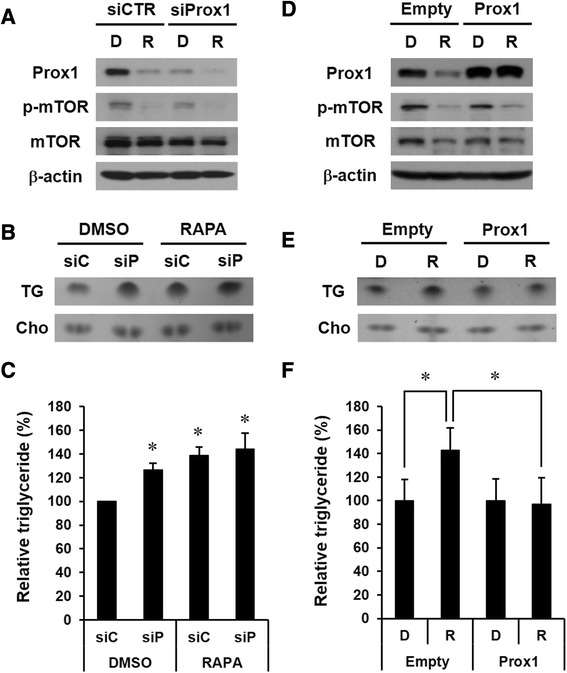
Prox1 plays a crucial role in the regulation of triglycerides in HepG2 cells. **a** HepG2 cells were transfected with siRNA against *Prox1* mRNA (siProx1) or control siRNA (siCTR). After 48 h, cells were treated with or without rapamycin for additional 48 h. Phosphorylated mTOR (p-mTOR) was detected to confirm the inhibitory effect of rapamycin. mTOR and β-actin were measured as quantitative controls. **b** Total lipids were harvested from HepG2 cells manipulated as (**a**). siC and siP indicate control siRNA and siProx1, respectively. TG: triglycerides, Cho: cholesterol. **c** The amount of relative triglycerides compared with cholesterol was also increased by siProx1 as well as rapamycin and was statistically significant (*p* < 0.05). **d** HepG2 cells were transfected with a plasmid expressing human Prox1 or an empty plasmid. After 24 h, cells were treated with or without rapamycin for additional 48 h. R and D indicate rapamycin and DMSO, respectively. **e** TLC analysis using (**d**) samples. TG: triglycerides, Cho: cholesterol. **f** The relative analysis shows that the increase in triglycerides caused by rapamycin was significantly down-regulated by Prox1 over-expression (*p* < 0.05)

To verify the role of Prox1 in the regulation of triglycerides, we employed siRNA against Prox1 mRNA (siProx1). siProx1 showed dramatic knock-down effect at 48 h after delivery, and the lipid analysis was performed at 4 days after siRNA. Prox1 expression was reduced by siProx1 as well as by rapamycin, and was almost negligible with rapamycin combined with siProx1 (Fig. 3a). The phosphorylated form of mTOR disappeared following the treatment with the inhibitor. After confirming the decrease of Prox1 expression by siRNA, we looked at the variation in triglycerides. Interestingly, the amount of triglycerides in HepG2 cells was increased by siProx1 (siP) as well as by rapamycin and was much higher with rapamycin combined with siProx1 (Fig. 3b). These results indicate that the amount of triglycerides in HepG2 cells is increased by the down-regulation of Prox1, which is similar to the result of rapamycin treatment. In comparison with intracellular cholesterol, the relative triglycerides were also significantly increased by siProx1 as well as by rapamycin (Fig. 3c). We next determined whether rapamycin-induced up-regulation of triglycerides was diminished by the compensation of Prox1 in HepG2 cells. We employed a Prox1 over-expression system and found that the down-regulation of Prox1 expression by rapamycin was reversed (Fig. 3d). Moreover, Prox1 over-expression down-regulated the increase of triglycerides induced by rapamycin but did not affect triglycerides in HepG2 cells in the absence of rapamycin (Fig. 3e and f). These data indicate that Prox1 plays a key role in the regulation of triglycerides and that the up-regulation of Prox1 may helpful for regulating triglycerides in hepatocytes treated with rapamycin.

### Rapamycin increases triglycerides but decreases Prox1 in mouse liver

**Fig. 4:**
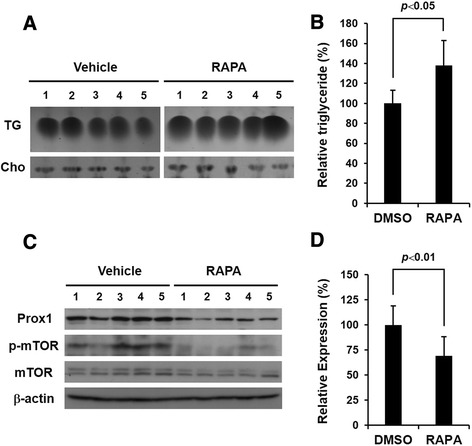
Prox1 in mouse liver is down-regulated by rapamycin. **a** Rapamycin or vehicle was injected daily into mouse (*n* = 5/group) intraperitoneally for 2 weeks. Total lipids and proteins were extracted from liver and used for TLC and western blot analysis, respectively. TG: triglycerides, Cho: cholesterol. **b** Relative triglycerides compared to cholesterol shows a statistically significant increase in quantity in rapamycin-treated tissues (*p* < 0.05). **c** Western blot analysis shows that rapamycin down-regulated prox1 expression. **d** The expression of prox1 was analyzed by comparing with β-actin, which shows a statistically significant down-regulation of prox1 expression by rapamycin (*p* < 0.01)

To understand the in vivo effect of rapamycin, we set out to evaluate the up-regulation of triglycerides and the down-regulation of Prox1 by daily intraperitoneal injections of rapamycin (4 mg/kg) into mouse. After 2 weeks, mice were sacrificed and total lipids and proteins were extracted from liver. Congruent with the results of the ex vivo experiment using HepG2 cells, rapamycin increased the amount of triglycerides but did not affect cholesterol in mouse liver (Fig. 4a). In comparison with cholesterol, relative triglycerides were significantly increased in rapamycin-treated liver cells (Fig. 4b). We next investigated the effect of rapamycin on the expression of Prox1 and found that rapamycin decreased the expression of Prox1 in mouse liver (Fig. 4c and d). The phosphorylated form of mTOR was detected for verifying the inhibitory effect of rapamycin, and mTOR or β-actin was detected as a quantitative control. These results indicate that rapamycin increases the amount of triglycerides while down-regulating the expression of Prox1 in mouse liver.

## Discussion

Rapamycin, an immunosuppressant, has an intracellular target, mTOR (FKBP12) and has been used to prevent graft rejection [8, 13, 14]. Although clinical application of rapamycin has been used successfully in transplantation, adverse effects can be provoked by its prolonged use. Hyperlipidemia including hypertriglyceridemia is one of the side effects of rapamycin [17–19]. Recent studies have reported that rapamycin can inhibit the transactivation of several transcription factors such as peroxisome proliferator-associated receptor-gamma (PPARγ) and CCAAT-enhancer-binding proteins (C/EBP), which play key roles in lipid metabolism [22] and it can change the expression of key enzymes required for fatty acid uptake and triglyceride synthesis in adipose tissue [23]. In spite of many trials to better understand the hyperlipidemia caused by rapamycin, the underlying mechanism remains unknown.

Prox1 is a transcription factor responsible for the development of several tissues including lymphatics and liver [3, 24]. Previous studies have mainly focused on the function of Prox1 in embryogenesis [1–3]. However, Prox1 is also known to regulate the activity of several nuclear receptors such as HNF4a (NR2A1) and LRH-1 (NR5A2), which indicates that Prox1 may play a role in the regulation of metabolism in the liver [5, 6]. Indeed, there is growing evidences that Prox1 participates in metabolism in addition to embryogenesis [25, 26]. However, whether Prox1 is directly related to lipid metabolism is currently unknown.

Because it has been well known that the prolonged use of rapamycin may cause unwanted side effects such as hyperlipidemia [17–21], we employed rapamycin to induce the up-regulation of triglycerides in HepG2 cells and investigated the effect of rapamycin on the expression of Prox1. Our data clearly show that rapamycin increased the amount of triglycerides in HepG2 cells but not cholesterol (Fig. 1b and c), which is similar to a previous report [14]. Although there have been several reports indicating the up-regulation of cholesterol by rapamycin in organ transplant patients [27], we did not find a significant increase of cholesterol in this study. We suppose that this discrepancy may arise from the difference in concentration or treatment time of rapamycin.

In our data, the down-regulation of Prox1 by rapamycin did not affect the proliferation of HepG2 cells (Fig. 1a), but a recent report showed that the treatment of rapamycin in lymphatic endothelial cells of lymphatic malformations (LM-LEC) diminished cellular proliferation [28]. This result may be caused by the physiological difference between hepatocytes and lymphatic endothelial cells. However, the role of Prox1 in cell physiology such as proliferation should be elucidated by scrutinizing the relationship between mTOR signaling and Prox1 using other hepatocyte cell lines or primary hepatocytes.

After confirming the up-regulation of triglycerides by rapamycin, we investigated the effect of rapamycin on the expression of Prox1. Interestingly, we found for the first time that rapamycin down-regulates Prox1 protein but does not affect its transcript (Fig. 2). This novel finding suggests the importance of mTOR signaling in maintaining the Prox1 protein in hepatocytes, which should be investigated further.

In order to understand the role of Prox1 in the regulation of triglycerides, we performed knock-down experiments using small-interfering RNAs (siRNAs), which mimic the effect of rapamycin in down-regulating the Prox1 protein. Interestingly, the amount of intracellular triglycerides was increased by the siProx1 alone, which is comparable with the effect of rapamycin, and was increased most by siProx1 and rapamycin synergistically (Fig. 3b and c). These data indicate that siProx1 can down-regulate the expression of Prox1, which is similar to the effect of rapamycin, and that Prox1 is a direct participant in the regulation of triglycerides in hepatocytes. The results of loss-of-function studies made us speculate whether the increased amount of intracellular triglycerides resulting from rapamycin is regulated by the replenishment of Prox1 through an over-expression system. As expected, the up-regulation of triglycerides by rapamycin returned to normal by Prox1 over-expression, which means that Prox1 is a crucial factor regulating triglycerides in hepatocytes (Fig. 3e and f). Next, we verified the effect of rapamycin on hepatocytes by systemic injections into mouse. After 2 weeks, lipids and proteins were extracted from mice livers. The results of the in vivo experiment showed that rapamycin increases the amount of triglycerides but down-regulates Prox1 expression in mouse liver (Fig. 4), which is comparable to the data using HepG2 cells.

Overall, we report here that rapamycin increased the amount of triglycerides and down-regulated the expression of Prox1 in HepG2 cells as well as in mouse liver. The knock-down and the over-expression experiments showed that Prox1 plays a critical role in the regulation of triglycerides in hepatocytes. From the results of this study, we conclude that mTOR signaling is important for maintaining the Prox1 protein responsible for the regulation of triglycerides in hepatocytes and suggest that this is one of reasons that triglycerides are up-regulated by rapamycin treatment. Further investigation should be performed to understand the relationship between mTOR signaling and the regulation of triglycerides by Prox1.

## Conclusions

In summary, our study suggests that rapamycin increased the amount of triglycerides and down-regulated Prox1 expression in hepatocytes. This result indicates that mTOR signaling is important for the maintenance of triglycerides by maintaining Prox1 expression.

## Methods

### Cell cultures, transfection and reagents

The HepG2 cell, a human hepatoma cell line, was purchased from the American Type Culture Collection (Manassas, VA). The cells were cultured in Dulbecco’s Modified Eagle Medium (DMEM) with 10 % fetal bovine serum (FBS), 5 % penicillin-streptomycin and 5 % sodium pyruvate and grown in an atmosphere containing 5 % CO_2_ at 37 °C. HepG2 cells were incubated with or without 10nM rapamycin (LC laboratories, MA) in low serum media (1 % FBS). HepG2 cells were transfected with 20nM of small interfering RNA (siRNA) against human *Prox1* (5′-GCAAAGAUGUUGAUCCUUCTT-3′, 5′-GAAGGAUCAACAUCUUUGCTT-3′) for knock-down experiment or an overexpression system (1 μg DNA) of Prox1 (human Prox1 coding sequence in pcDNA3, kindly provided by Prof. Y-K Hong, University of Southern California) for expressional compensation using Lipofector-2000 (Aptabio, Korea). 20 μg/ml of cycloheximide (CHX, Sigma-Aldrich, MO) was used to chase the stability of Prox1 protein. After incubation for 12 h with or without rapamycin, HepG2 cells were treated with cycloheximide from 12 min to 6 h and lysed for western blot analysis.

### Cell proliferation assay

The cell proliferation assay was performed using a Premixed WST-1 Cell Proliferation Assay kit (TaKaRa, Japan), as described previously [29]. In brief, HepG2 cells (2 × 10^4^ cells/well) were seeded in 24-well plates (SPL Inc., Korea) and cultured overnight. Media were then replaced with fresh media (1 % FBS) containing rapamycin. Cells were then allowed to grow for 48 h. WST-1 reagent was added to each well and cells were incubated for an additional 4 h. Optical absorbance was subsequently measured at a wavelength of 450 nm using a microplate reader (Molecular Devices, CA) and the cell proliferation count was estimated based on a standard curve that was prepared in parallel.

### Thin layer chromatography (TLC) analysis

Total lipids were extracted from cells (1 × 10^6^) or liver tissues (50 mg) using the chloroform:methanol (1:2 v/v) extraction method reported by Bligh and Dyer [30]. Triglycerides and cholesterol were separated using a TLC silica gel 60 plate (Merck, Germany) in a horizontal TLC chamber with an 9 cm separation length with a solution of chloroform:methanol:water (60:30:5 v/v/v). An additional separation from the origin to an 18-cm separation length was achieved with a hexane:diethyl ether:acetic acid (80:20:1.5 v/v/v) solution. TLC plates were visualized by spraying with H_2_SO_4_, followed by heating at 130 °C for 30 min. Acquired images were processed and analyzed using the Image J program.

### Reverse transcription-polymerase chain reaction (RT-PCR)

The expression of Prox1 transcripts were analyzed by semi-quantitative RT-PCR with human *Prox1*-specific primers (sense: 5′-GCAAGTTGTGGACACTGTGGT-3′, anti-sense: 5′-GGCAGACTGGTCAGAGGAGTT-3′) and *glyceraldehyde 3-phosphate dehydrogenase* (*GAPDH*)-specific primers (sense: 5′-AGAAGGCTGGGGCTCATTTG-3′, anti-sense: 5′-AGGGGCCATCCACAGTCTTC-3′) to achieve equal amounts of cDNA templates. Total RNA was isolated from HepG2 cells using TRIzol reagent (Life Technology, CA) and reverse-transcribed using M-MLV reverse transcriptase (Enzynomics, Korea) and 15 mer oligo-dT (Promega, WI). Using cDNA as a template, PCR was performed using the following protocols: (1) 94 °C for 5 min; (2) 26 cycles at 94 °C for 30 s, 55 °C for 30 s, and 72 °C for 1 min; and then (3) 72 °C for 10 min before cooling to 4 °C. Ten microliters of PCR products were loaded on a 1.5 % agarose gel containing ethidium bromide and visualized by ultraviolet irradiation. Quantitative real-time PCR analysis was performed using QuantiSpeed SYBR Green kit (PhileKorea, Korea) as follows: 50 °C for 10 min, 95 °C for 2 min, and 40 cycles of 95 °C for 10 s, and 55 °C for 30 s.

### Protein extraction and Western blot analysis

Cell lysates were prepared using RIPA buffer (50 mM Tris-HCl [pH 7.4], 150 mM NaCl, 1 % Nonidet P-40, 0.1 % sodium dodecyl sulfate [SDS] and 0.5 % sodium deoxycholate) containing protease and phosphatase inhibitors (Sigma-Aldrich). Samples were quantified using the Bradford Protein Assay kit (Pierce Biotechnology, IL) according to the manufacturer’s instructions. Then 20–30 μg of proteins were separated on 10 % SDS polyacrylamide gel electrophoresis and transferred to polyvinylidene difluoride membranes (PVDF; GE Healthcare, UK). Western blots were performed with anti-Prox1 antibody (rabbit polyclonal, kindly provided by Prof. Y-K Hong, University of Southern California), anti-phospho-mTOR antibody (sc-101738, Santa Cruz Biotechnology, CA), anti-β-actin antibody (sc-47778, Santa Cruz Biotechnology), appropriate secondary antibodies and an enhanced chemiluminescence detection reagent (Amersham Pharmacia Biotech, NJ).

### Rapamycin injection into mouse and sample preparation

Eight-week-old BALB/c mice (all male, *n* = 5/group) were purchased from RaonBio (Korea) and daily injected intraperitoneally with rapamycin (4 mg/kg) or vehicle (5 % polyethylene glycol 400 [Sigma-Aldrich] and 5 % Tween 80 [Sigma-Aldrich]) as described previously [31]. After 2 weeks, mice were sacrificed and total proteins or lipids were extracted from mouse liver. The same weight of liver tissue was lysed using RIPA buffer containing protease/phosphatase inhibitors (Sigma-Aldrich) and 50 μg of total protein was used for western blot analysis. For TLC analysis, total lipids were extracted from 50 mg of liver tissue using chloroform:methanol (1:2 v/v) solution. All surgical and experimental procedures were performed according to the guidelines of the Animal Care and Use Review Committee of Hoseo University, Korea.

### Statistical analysis

Statistical analysis was performed using GraphPad Prism 5.0 (GraphPad Software Inc., La Jolla, CA). Results were expressed as mean ± standard error of three independent experiments. One-way analysis of variance and Tukey’s test were used to detect differences, with *P* < 0.05 and *P* < 0.01 considered significant.

## Abbreviations

mTOR: mammalian target of rapamycin
Prox1: prospero-related homeobox 1
RT-PCR: reverse transcription-polymerase chain reaction
siRNAs: small-interfering RNAs
TLC: thin layer chromatography
